# Characterisation of *Fusarium oxysporum* f. sp. *radicis*-*lycopersici* in Infected Tomatoes in Inner Mongolia, China

**DOI:** 10.3390/jof10090622

**Published:** 2024-08-30

**Authors:** Yongqing Yang, Yong Wang, Jing Gao, Zhidan Shi, Wenjin Chen, Haiyan Huangfu, Zhengnan Li, Yan Liu

**Affiliations:** 1 Inner Mongolia Academy of Agricultural & Animal Husbandry Sciences, Hohhot 010031, China; yyq2022@outlook.com (Y.Y.); wangyonging@outlook.com (Y.W.); gaojing1204@126.com (J.G.); zhidan_s@outlook.com (Z.S.); chenchen416526@outlook.com (W.C.); huangfuhaiyan@sohu.com (H.H.); 2 College of Horticulture and Plant Protection, Inner Mongolia Agricultural University, Hohhot 010018, China

**Keywords:** identification of fusarium crown and root rot, *Fusarium oxysporum* f. sp. *radicis-lycopersici*, fungicide sensitivity, plant resistance, Inner Mongolia

## Abstract

Fusarium crown and root rot (FCRR), caused by *Fusarium oxysporum* f. sp. *radicis-lycopersici* (FORL), is an economically important disease that affects tomatoes worldwide and has become more prevalent in China in recent years. In 2021 and 2022, tomato plants in greenhouses in Hohhot, Inner Mongolia, were observed showing symptoms of stunting, premature loss of lower leaves, and root rot. Fungal pathogens were isolated from 20 infected tomato plants and identified based on morphological observation and DNA sequencing. Twelve isolates were consistently identified as *Fusarium oxysporum* f. sp. *radicis*-*lycopersici* (FORL) via an analysis of the *ITS*, TEF-*1α*, and *pgx4* genes. This is the first report of FORL in Inner Mongolia, China. The isolates were examined for their pathogenicity by inoculating them on tomatoes, eggplants, peppers, and chickpeas. The fungicide sensitivity of the isolates was determined. Effective concentrations for 50% growth inhibition (EC_50_) were measured using seven fungicides. The EC_50_ values of tebuconazole and prochloraz were <1.0 μg·mL^−1^, exhibiting the most effective inhibition among the fungicides tested. Additionally, FORL resistance screening of tomato germplasms was performed. One tomato variety was resistant to FORL, and the remaining 43 germplasm lines showed various levels of resistance. The rates of highly susceptible, moderately susceptible, susceptible, and moderately resistant germplasms accounted for 29.55%, 22.73%, 40.91%, and 4.55% of the 44 germplasms tested, respectively.

## 1. Introduction

Fusarium crown and root rot (FCRR), caused by *Fusarium oxysporum* f. sp. *radicis-lycopersici* (FORL), is a severe soil-borne fungal disease that affects tomato production worldwide. The disease was first reported in Japan in 1974 and has since been found in 32 countries, including China [1]. The disease spreads rapidly in greenhouse cultivation areas and causes increased losses in continuous cropping areas [2]. China is the world’s largest tomato producer, with a total tomato production of 487.49 million tonnes in 2020 [3]. Since its first report in Beijing greenhouses in 2007 [4], FCRR has been reported in many areas, including Shandong, Jiangsu, Gansu, Xinjiang, Ningxia, Qinghai, and Liaoning—the major tomato production areas in China [5]. Disease incidence was as high as 80% in Shouguang greenhouses (Shandong Province), with a mortality rate of 30%, leading to severe yield losses [6].

The primary symptoms of FCRR include dark brown lesions at the soil–plant stem junction and vascular browning, leading to wilting and dying at the 3–5-leaf stages or shrivelling and dark brown discoloration at the stem base of mature plants. The optimal temperature for FORL to cause symptoms is 18 °C. Its host range includes 37 plant species, including 10 plant species within the *Solanaceae*, *Leguminosae*, *Cucurbitaceae*, and *Chenopodiaceae* families, which exhibit high levels of susceptibility [4,7,8].

The long-distance dispersal of the pathogen occurs via the movement of infected plants and contaminated seeds. The pathogen infects the host without specific infection sites (e.g., root tips or wounded tissue) and does not form specialised structures such as appressoria [9]. Given that the pathogen is a soil inhabitant and causes significant economic losses, the most environmentally friendly and effective option for controlling this disease is the development of resistant cultivars. To date, there have been very few releases of new tomato cultivars with FORL resistance that are commercially competitive in China [10,11]. Therefore, there is an urgent need to select and provide high-quality, highly resistant tomato germplasms to accelerate the breeding of FORL-resistant varieties.

Tomatoes are important vegetables in Inner Mongolia. In recent years, the incidence of soil-borne diseases has increased because of continuous monocropping, which has become a constraining factor in the development of the tomato industry. The aim of this study was to confirm the establishment of FORL in Hohhot, Inner Mongolia, and characterise it through fungicide sensitivity assays. Resistance evaluations of 44 tomato germplasms against the disease were also performed to provide a basis for disease prevention and resistance breeding programmes.

## 2. Materials and Methods

### 2.1. Pathogen Isolation

**Figure 1:**
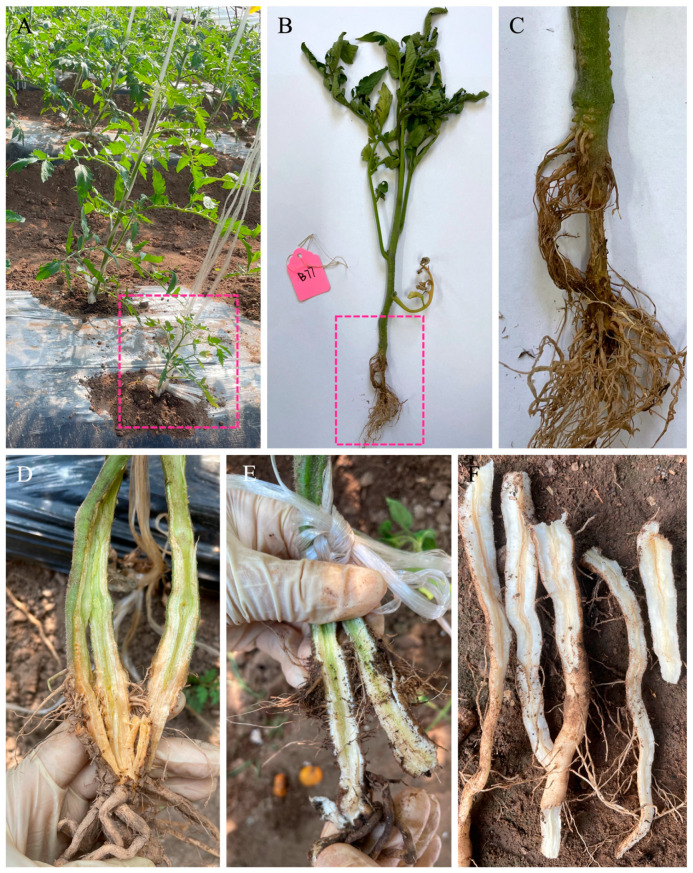
Symptoms of Fusarium crown and root rot caused by *Fusarium oxysporum* f. sp. *radicis*-*lycopersici* on tomato plants in fields: (**A**) Symptoms in the infected tomato seedlings included stunting, yellowing, and premature loss of lower leaves in August 2021. (**B**,**C**) Dark brown lesions and root rot were observed on the taproot and lateral root of the plant (pink dashed box) in panel A. (**D**) Symptoms of root rot were observed in mature plants in July 2022, with reddish-to-brown discoloration of the root and stem. (**E**) Healthy roots and stem. (**F**) Dissected taproots of infected plants also developed brown lesions, as seen when sliced lengthwise.

Twenty infected tomato plants were collected from greenhouses in Hohhot, Inner Mongolia, China, in 2021 and 2022. The symptoms observed included stunting, premature loss of lower leaves, and crown rot in young tomato plants, along with reddish-to-brown discoloration of the root and stem when sliced lengthwise in mature plants (Figure 1).

Tomato roots were washed with tap water to remove soil particles and then dried on sterile filter paper. Segments (0.5 to 1.0 cm) were cut out from the diseased tissues and subsequently disinfected in 2% sodium hypochlorite solution for 30 s and 75% ethanol for 1 min, followed by rinsing three times with sterile distilled water, after which they were placed on potato dextrose agar (PDA) in plastic Petri dishes with a diameter of 9 cm, and then incubated at 25 °C in the dark for 4 to 5 days. The isolates were purified using the single-spore method.

### 2.2. Morphological Identification of Pathogens

Morphological characteristics of the colonies and spores were observed by culturing the purified isolates on potato dextrose agar (PDA) and mung bean culture agar at 25 °C in the dark for 4 to 5 days. Additionally, the isolates were inoculated in potato dextrose broth (PDB) and incubated at 25 °C on a shaker at 150 rpm for 7 days for microscopic observation. The spore morphology of the isolates was observed using a compound microscope (ECLIPSE NI-U^®^, Nikon, Tokyo, Japan) fitted with a digital camera (Y-TV55^®^, Nikon, Tokyo, Japan).

### 2.3. Molecular Identification of Pathogen

The fungal isolates were cultured in a PDB medium at 25 °C on a shaker at 150 rpm for 5 days. The mycelia were collected and washed twice with sterile distilled water. The total DNA was extracted using an EasyPure Genomic DNA Extraction Kit (TransGen, Beijing, China), following the manufacturer’s instructions.

The internal transcribed spacer region (ITS) of the fungi was amplified by polymerase chain reaction (PCR) with the primer pair ITS1 (5′-TCCGTAGGTGAACCTGCGG-3′) and ITS4 (5′-TCCTCCGCTTATTGATATGC-3′). A region of the translation elongation factor gene (*TEF-1α*) was amplified using the primer pair ef1 (5′-ATGGGTAAGGAGGACAAGAC-3′) and ef2 (5′-GGAAGTACCAGTGATCATGTT-3′). The amplification conditions were as follows: 95 °C for 2 min; 35 cycles of 94 °C for 40 s, 58 °C for 30 s, and 72 °C for 1 min, followed by a final extension at 72 °C for 5 min. Additionally, the exopolygalacturonase gene *pgx4* was amplified using the primer pair sprl (5′-GATGGTGGAACGGTATGACC-3′) and sprlr (5′-CCATCACACAAGAACACAGGA-3′), following previously described PCR conditions [12]. The PCR products were Sanger sequenced at BGI Tech Solutions (Beijing Liuhe) Co. Ltd., Beijing, China. DNA sequences were analysed using the BLASTn algorithm in the GenBank database. Representative TEF*-1α* and *pgx4* gene sequences with high similarity to *Fusarium oxysporum* were selected and combined to construct a phylogenetic tree using the neighbour-joining tree build method with a bootstrap test (1000 replicates) in Geneious Prime 2024.0.2.

### 2.4. Pathogenicity Assay

To test the pathogenicity to tomato plants, the fungal isolates were cultured on PDA for 5 days and then transferred into a 500 mL flask containing 100 mL of PDB, which was incubated at 25 °C in a shaker at 150 rpm for 3 days. To prepare the inoculum, the culture suspension was filtered through four layers of sterile gauze to remove mycelia and was then adjusted with sterile distilled water to obtain 1 × 10^7^ spores·mL^−1^.

‘Moneymaker’ and ‘B77′ tomato seedlings used for the pathogenicity tests were planted in open, flat seed trays filled with sterilised commercial potting mixes containing peat, vermiculite, and perlite. The seedlings were grown in a greenhouse at the Inner Mongolia Academy of Agricultural and Animal Husbandry Sciences between April and May 2022, under ambient environmental conditions. When the seedlings reached the 3–4-leaf stages, the roots were washed with water and artificially bruised to produce slight damage before being transplanted into prepared sterile soil, followed by pouring 30 mL of the pathogen spore suspension around the roots [13]. The experiment was repeated twice, with 10 seedlings inoculated each time, and they were kept at 20 ± 2 °C with 16 h of light and 8 h of darkness.

To test pathogenicity to other plants in the families Solanaceae and Leguminosae, seedlings of chilli pepper (*Capsicum frutescens* L.) and eggplant (*Solanum melongena* L.) at the 4–5-leaf stages were obtained from a commercial market; the chickpea (*Cicer arietinum* L.) cultivar ‘Xinying 3′ at the 7–8-leaf stages was provided by the Food Legumes Breeding and Cultivation Research Laboratory of the Inner Mongolia Academy of Agricultural and Animal Husbandry Sciences in April 2022. The experiment was repeated twice, with 10 seedlings inoculated each time, and the inoculation was performed as described above.

After 30 days, symptom development in all seedlings was observed and assessed according to the method reported in [7]. Re-isolation of the pathogens was conducted when the inoculated roots developed symptoms according to the isolation methods described above (Section 2.1).

### 2.5. Fungicide Sensitivity Assay

Sensitivity to seven fungicides was determined using a radial growth assay. Seven active ingredients—hymexazol, tetramethylthiuram disulphide, pyraclostrobin, azoxystrobin, tebuconazole, prochloraz, and thiophanate-methyl (aladdin, Shanghai, China)—were used to evaluate sensitivity. Next, 5 mm plugs from the edge of 7-day-old isolates—the two isolates T36-1f and T38-2c, which exhibited a high disease incidence rate—were inoculated onto the centres of Petri dishes (9 cm) containing PDA medium formulated with a range of concentrations. The final concentrations of the fungicides in the PDA are provided in Table S1. The technical-grade fungicides available as powders were dissolved in dimethyl sulfoxide (DMSO) to make the initial concentrations of solution and then added to PDA cooled to 50 °C. Colony diameters were measured in two perpendicular directions 7 days after inoculation, and the tests were repeated three times. The half-maximal effective concentration (EC_50_) values were calculated as previously described [14,15], and statistical analysis was performed using Microsoft Excel software (Version 16.88).

### 2.6. Resistance of Tomato Germplasms

To determine the resistance of tomato germplasms to the pathogen, 44 tomato germplasm lines were assessed; these lines were provided by the Tomato Breeding and Cultivation Research Group of the Inner Mongolia Academy of Agricultural and Animal Husbandry Sciences and included 33 breeding lines and 11 commercially available varieties (see Table S2). A spore suspension of isolate T36-1f was inoculated on the plant materials. Disease was observed 30 days after the inoculation. The experiment was repeated twice, with 10 seedlings inoculated each time. The disease severity scale was calculated as described in the referenced literature [13].

## 3. Results

### 3.1. Morphological Identification

**Figure 2:**
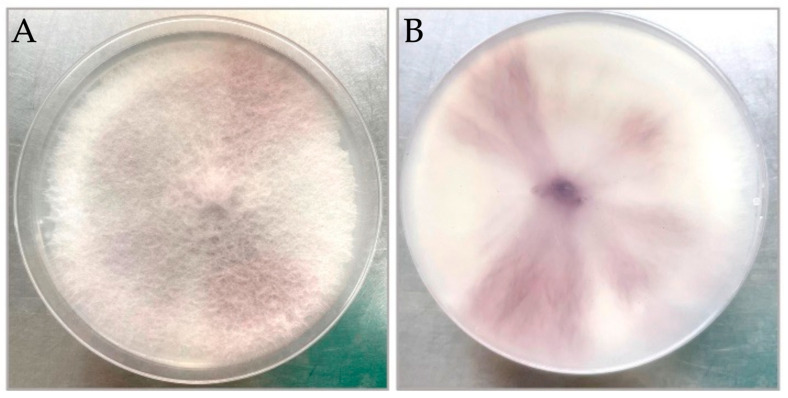
Colonies of isolate T36-1f of FORL on PDA after 7 days of incubation at 25 °C: (**A**) top view; (**B**) back view.

**Figure 3:**
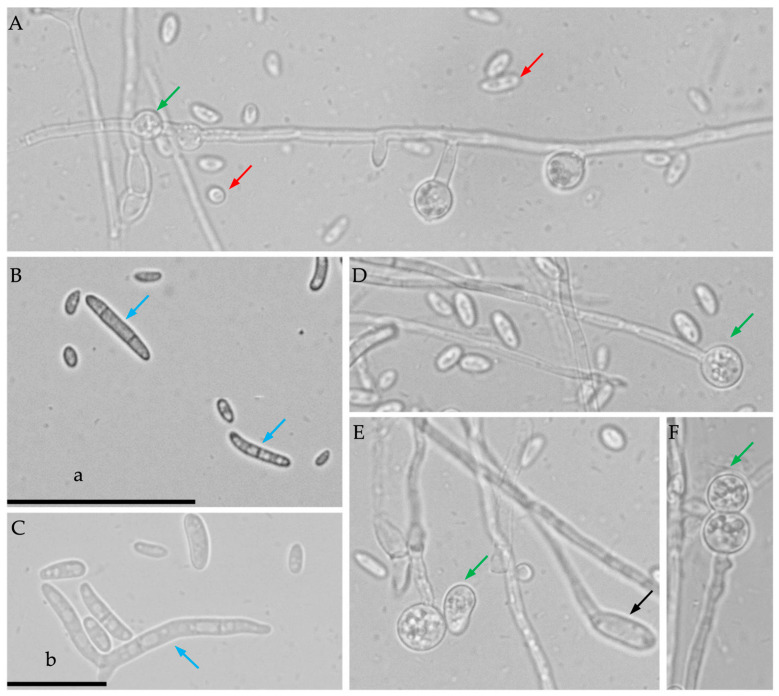
Morphologies of *Fusarium oxysporum* f. sp. *radicis*-*lycopersici* isolate T36-1f in this study: (**A**) non-septate, ovoid-to-elliptical microconidia (red arrow) and intercalary chlamydospores (green arrow) were formed; (**B**,**C**) 1 to 3 septate, sickle-shaped macroconidia (blue arrows); (**D**) terminal chlamydospores (green arrow); (**E**,**F**) catenate chlamydospores (green arrows) were formed on conidial hyphae, with a spherical-to-elliptical shape, and conidiophore (black arrow). a: the scale bar is 50 μm in panel (**B**); b: the scale bar is 20 μm in panels (**A**,**C**–**F**).

Fungal colonies of the isolates grown on PDA reached a diameter of 8 cm after 7 days of incubation at 25 °C, exhibiting aerial mycelia that were white, medium density, round, and with purple pigment observed on the back view (Figure 2). The pathogen produced abundant non-septate, ovoid-to-elliptic microconidia on both PDA and PDB media (Figure 3A), and one to three septate, sickle-shaped macroconidia on mung bean agar (Figure 3B,C). On the PDB, terminal, intercalary, or catenate chlamydospores were formed on the conidial hyphae and were mostly spherical to elliptical (Figure 3A,D–F). Twenty-three isolates were obtained from infected plant tissues, twelve of which were consistently identified as *Fusarium* species based on their microconidial and macroconidial characteristics on PDA. Moreover, the other eleven isolates were identified, including five isolates of *Plectosphaerella cucumerina*, one isolate of nonpathogenic *F*. *oxysporum*, one isolate of *F. equiseti*, three isolates of *Pythium aphanidernatum*, and one isolate of *Aspergillus spp*.

### 3.2. Molecular Identification

**Figure 4:**
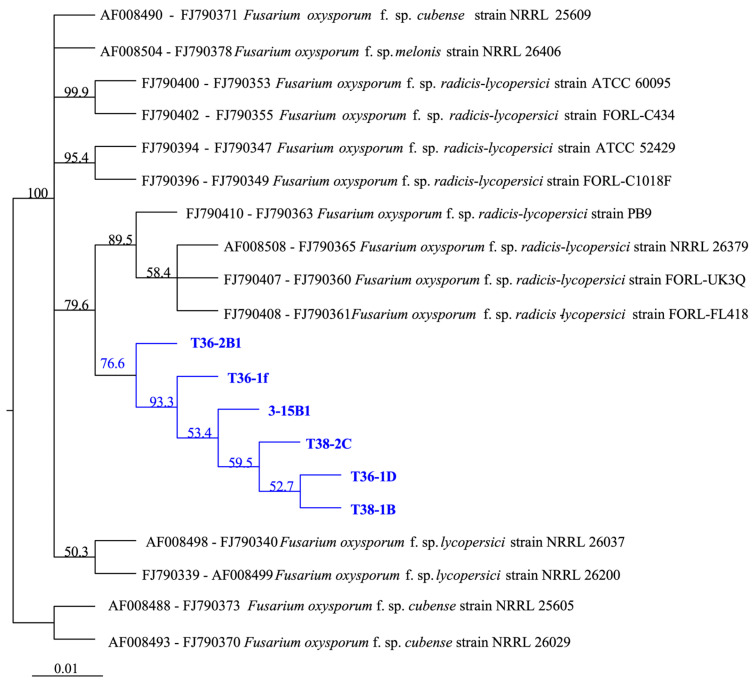
Phylogenetic tree established using the combined DNA sequences of the elongation factor 1α (TEF*-1α*) and the exopolygalacturonase gene (*pgx4*). The sequences were aligned using ClustalW with MEGA11.0 and the tree was developed using the neighbour-joining tree build method with bootstrap test (1000 replicates) in Geneious Prime 2024.0.2. Isolates highlighted in blue were the FORL isolates obtained from infected tomato plants in this study.

Molecular identification of 12 isolates was performed by analysing the DNA sequences of the 16S rDNA region, the elongation factor 1α (TEF-*1α*), and the exopolygalacturonase gene (pgx4), which generated fragments of approximately 530 bp, 750 bp, and 900 bp, respectively. The GenBank Accession Numbers for genes sequenced from six representative isolates—T36-1f, T36-2B1, T36-1D, T38-2C, T38-1B, and 3-15B1—are provided in Table 1. The amplified fragment of the 16S rDNA region had 100% identity homology with 12 isolates and those of *F. oxysporum*, e.g., MG736729 isolated from Shandong and MK212364 isolated from Gansu, China, demonstrating that the isolates were identified as *F. oxysporum*. The similarity of the elongation factor 1α (TEF-*1α*) and the exopolygalacturonase gene (pgx4) among isolates was more than 99.5%. A phylogenetic tree based on concatenating the TEF-*1α* and pgx4 genes confirmed that the isolates obtained in this study clustered together with FORL sequences sequenced from strains NRRL 26379, FORL-UK3Q, FORL-FL418, and PB9 (Figure 4). The results showed that the ITS sequences of the 12 isolates were 100% identical and the similarity of the elongation factor 1α (TEF-*1α*) and the exopolygalacturonase gene (*pgx4*) among isolates was more than 99.5%. Therefore, six representative isolates, T36-2B1, T36-1D, T36-1f, T38-1B, T38-2C, and 3-15B1, were included in establishing the phylogenetic tree.

### 3.3. Pathogenicity Test

**Figure 5:**
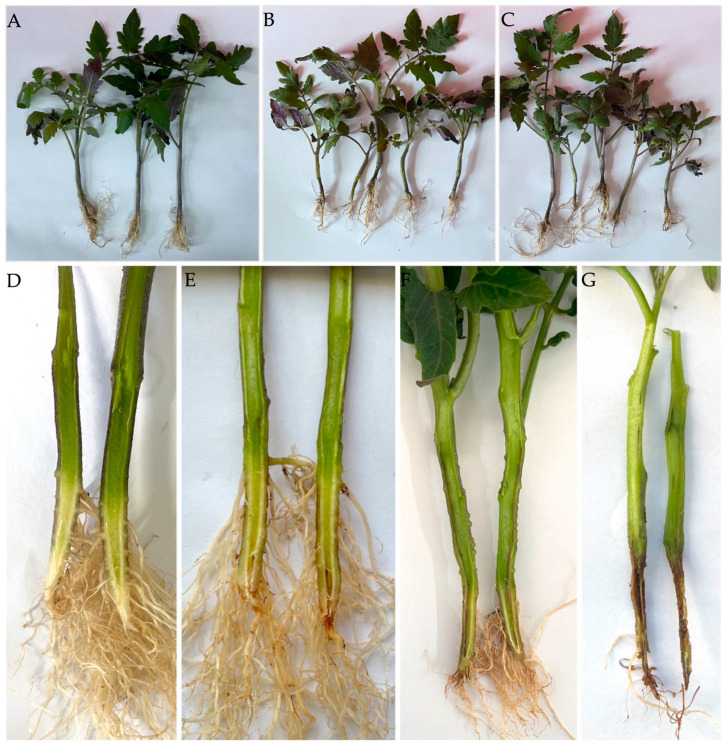
A pathogenicity test of isolates of *Fusarium oxysporum* f. sp. *radicis*-*lycopersici* (FORL) to tomato seedlings: (**A**) An uninoculated control. (**B**,**C**) Dark brown lesions were observed on the taproot and lateral root, and the whole stem base became girdled and constricted by dark brown lesions in the plants inoculated with FORL strains T36-2B1 and T36-1f, respectively; (**D**) A vascular bundle in an uninoculated plant. (**E**–**G**) Dark brown lesions developed in the vascular bundle in inoculated plants with different degrees of disease severity.

Twelve isolates identified as *Fusarium oxysporum* f. sp. *radicis*-*lycopersici* were used to inoculate 3–4-leaf tomato seedlings, and two strains of them were used to inoculate 4–5-leaf peppers and eggplants. After 30 days of inoculation, the disease incidence was 100% for the twelve strains; furthermore, the symptoms that developed on tomato seedlings were similar to those observed in the field, including brown and dark lesions that constricted the stem base. Moreover, dark brown lesions developed and were observed in the vascular bundle in inoculated plants. The tomato seedlings inoculated with isolates T36-2B1 and T36-1f are shown in Figure 5 as representative examples. The pathogens were re-isolated from the diseased roots and exhibited the same morphological traits as the original isolates used for inoculation. Pathogenicity tests indicated that the isolates caused disease in tomatoes, confirming Koch’s postulates.

**Figure 6:**
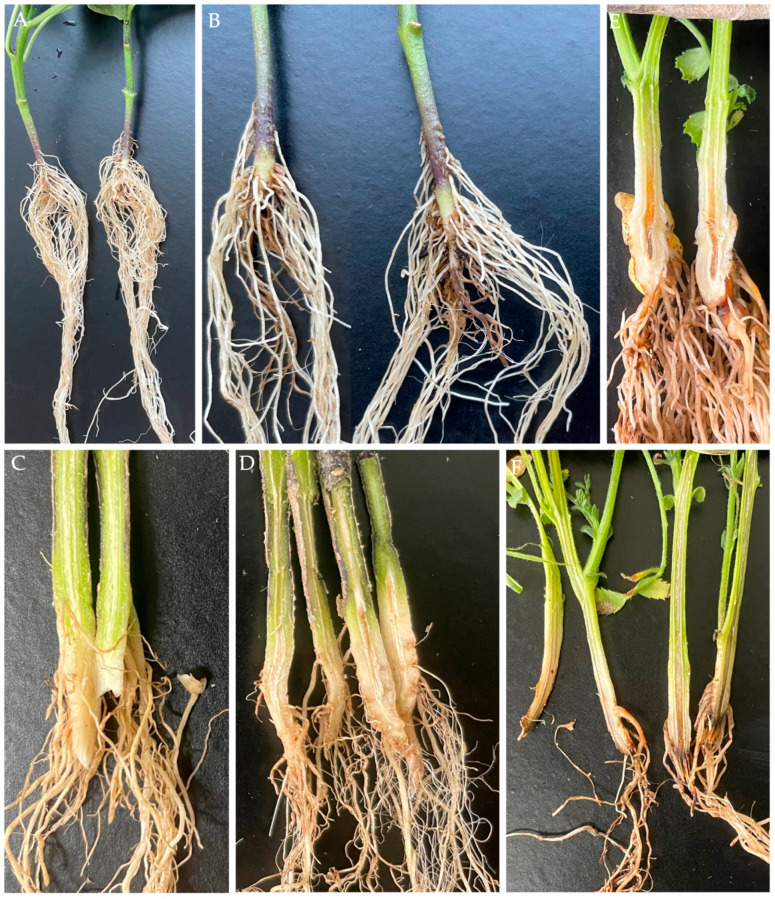
The effects of inoculation with *Fusarium oxysporum* f. sp. *radicis-lycopersici* isolate T36-1f on chilli pepper, eggplant, and chickpea plants: (**A**) Brown lesions developed on the taproots and lateral roots of inoculated chilli pepper. (**B**) An uninoculated chilli pepper. (**C**) Brown discoloration of the roots and stems of inoculated eggplants was observed when sliced lengthwise. (**D**) Uninoculated eggplants. (**E**) Uninoculated chickpea. (**F**) Symptoms of root rot and necrosis that developed through the main root in chickpea.

In addition, artificially inoculating chilli peppers and chickpeas with isolates T36-1f and T36-2B1 produced brown lesions, discoloration of the roots and stems, and necrosis in the vascular bundle of the chickpeas (Figure 6A,B,E,F). The lesions were small in eggplants, with only a slight discoloration of the roots (Figure 6C,D). Re-isolation of the pathogens from the inoculated plants was carried out and confirmed using the original strains (Table 2). The results demonstrate that isolates T36-1f and T36-2B1 could infect chilli peppers, eggplants, and chickpeas.

### 3.4. Sensitivity of FORL Isolates to Fungicides

The EC_50_ values of the seven fungicides against isolates of FORL T36-1f and T38-2C were determined via toxicity tests. The results (Table 3) suggested the seven tested fungicides exhibited varying degrees of mycelial growth inhibition against the isolated FORL strains. The EC_50_ values of tebuconazole and prochloraz were <1.0 μg·mL^−^^1^, exhibiting the most effective inhibition, followed by pyraclostrobin and azoxystrobin, with EC_50_ values less than 5.0 μg·mL^−^^1^. Tetramethylthiuram disulphide and thiophanate-methyl, with EC_50_ values between 9.490 μg·mL^−^^1^ and 15.142 μg·mL^−^^1^, showed relatively moderate inhibition against the strains. In contrast, hymexazol, which has broad-spectrum activity against various plant pathogens, was less effective than the other fungicides tested against the strains, with EC_50_ values > 100.0 μg·mL^−^^1^.

### 3.5. Resistance Screening in Tomatoes

To evaluate the resistance of tomato germplasm accessions and cultivars to FORL, the disease index of 44 tomato entries was calculated after 30 days of inoculation, based on the method described previously [13,16]. Among the 33 breeding materials, the hybrid variety ‘Nei Fan 401′ was identified as resistant to FORL, whereas two lines showed moderate resistance (Table 4). Thirteen lines were highly susceptible, including one yellow tomato line (B66); eighteen entries were susceptible. Among the eleven commercial tomato varieties, nine varieties showed moderate susceptibility, and two varieties were susceptible. Overall, 29.55% of the entries were highly susceptible, 40.91% were susceptible, 22.73% were moderately susceptible, 4.55% were moderately resistant, and 2.27% were resistant. No immune variety was detected in the present study.

## 4. Discussion

Through morphological observation, along with molecular and genetic analyses, we identified FORL that causes FCRR in Inner Mongolia, China. The colony morphology of the isolates in the present study exhibited high similarity to that of the previously described FORL isolate MAF103007 [17] and isolates from greenhouses in Shandong, China [18,19]. Nevertheless, this is the first report of FORL in tomato greenhouses in Hohhot, Inner Mongolia. Interestingly, five isolates of *Plectosphaerella cucumerina* were also obtained and identified in the samples infected by *Fusarium spp.* in the present study. The interaction of the two species and their invasion mechanisms might be a novel aspect for understanding FORL in further research.

Molecular and genetic analyses are commonly employed as effective ways to identify *Fusarium* spp., especially to detect morphologically indistinguishable *formae speciales*. *F. oxysporum* f. sp. *radicis*-*lycopersici* was determined as a new formae specialis [20], rather than a new race of *F. oxysporum* f. sp. *lycopersici* that is the causal agent of Fusarium wilt, based on differences in symptoms and host specificity [4,8]. For the identification of *F. oxysporum* f. sp. *radicis*-*lycopersici*, studies have reported the use of conserved fungal primers (e.g., ITS1 and ITS4) and specialised primers for housekeeping genes (e.g., the translation elongation factor 1α) for conventional PCR amplification [16,21], allowing the identification of *F. oxysporum* species. A gene encoding a pathogenicity trait, exopolygalacturonase (*pgx4*), was amplified and analysed to distinguish formae speciales within *F. oxysporum* [12,22,23]. A novel KASP-SNP detection technology for identifying FORL and FOL was established in 2022, achieving a 100% positive detection rate for FORL and FOL physiological races 1 and 2 [24]. The mating type, elongation factor-1*α*, and exopolygalacturonase sequences were employed to address the evolutionary relationships between different isolates of the *F. oxysporum* species complex, with a special emphasis on the formae speciales *lycopersici* and *radicis*-*lycopersici* [18]. Therefore, the TEF*-1α* and *pgx4* genes were employed and combined to construct the phylogenetic tree, illustrating that the isolates obtained were FORL. Moreover, the *SIX* gene amplifications (*SIX1*–*SIX7*) were conducted following the method described in [25], and the results showed that the amplicons of the *SIX1*, *SIX2*, *SIX3*, *SIX4*, and *SIX5* genes were not obtained, indicating that *SIX1*–*SIX5* were not exclusively present in all of the isolates in this study, and confirming the identification of FORL.

Given that *F. oxysporum* can survive for more than 10 years in the soil and spread through water, tools, and equipment in the field, effective control strategies against FORL include soil treatment before transplanting and synthetic fungicide application during the growing season. Although the inhibitory efficacy of fumigants against *F. oxysporum* f. sp. *radicis*-*lycopersici* was not addressed, this study evaluated the antifungal activity of nine fumigants against *F. oxysporum* f. sp. *lycopersici* and demonstrating that, at 20 mg/L, the radial growth of the pathogen was inhibited 100% by formaldehyde and >80% by phosphine [26]. On the other hand, fungicide application is one of the effective treatments to inhibit *F. oxysporum*, as reported. Prochloraz and bromuconazole were the most effective fungicides against the pathogen both in vitro and in vivo [27]. A previous study revealed that tebuconazole + trifloxystrobin (at 1000 ppm) completely inhibited the growth of *F. oxysporum* f. sp. *lycopersici* (*Fol*) in field experiments [28]. Similarly, the fungicides Nativo (tebuconazole 50% + trifloxystrobin 25%) and carbendazim are effective against *Fol* at 750 and 1000 ppm concentrations [29]. Another study in India also demonstrated that tebuconazole 50% + trifloxystrobin 25% WP exhibited the most efficacy against *Fol*, followed by tebuconazole 250 E.C and azoxystrobin 23% SC [30]. The in vitro evaluation of fungicide sensitivity in the present study, illustrating that pyraclostrobin, tebuconazole, and prochloraz are highly effective for inhibiting the mycelial growth of FORL, was consistent with the findings of previous studies. It has been demonstrated that pyraclostrobin, propiconazole, and tebuconazole can be used to control tomato crown and root rot in fields, and pyraclostrobin at 60 mg a.i./plant exhibits the highest control efficacy [31,32]. The difficulty of treating soil-borne pathogens with fungicides in fields with high levels of *F. oxysporum* soil inoculum is well known. Therefore, according to the results of this study, the evaluations of fungicides (e.g., prochloraz, tebuconazole, pyraclostrobin, azoxystrobin, and hymexazol) in vivo (including the application time of the fungicides) need to be conducted and addressed in future studies, which may be included in formulating management strategies for the disease FCRR.

Breeding disease-resistant varieties is another way to control this disease effectively. The single dominant gene *Frl*, located on chromosome 9, has been confirmed in Peruvian tomato germplasm [33]. A recent study showed that the gene *Frl* is mapped to chromosome 1 [34]. Additional potential genetically resistant sources were identified by analysing the transcriptome data for FORL-resistant (cv. ‘19912′) and FORL-susceptible (cv. ‘Moneymaker’) tomato cultivars [35,36]. Additionally, a study demonstrated that overexpression of the lectin receptor kinase gene *SlLecRK1* substantially increased the resistance of the susceptible tomato cultivar ‘Moneymaker’ to FORL [37]. The present study identified ‘Nei Fan 401′ as a resistant variety, which is a hybrid cultivar that also exhibited favourable agronomic characteristics such as good fruit quality, desirable flavour, and greater adaptability in North China. There is a need to further study resistance mechanisms to FORL, and the ‘Nei Fan 401′ tomato could be a good source of genetic resistance genes for breeding. Combined with the host specificity test, the pathogen can cause symptoms in chilli peppers, eggplants, and chickpeas, indicating that FORL has a broad host range [7]. Therefore, in greenhouse tomato cultivation, once FORL occurs, it is advisable to rotate crops and avoid planting eggplants and peppers, so as to reduce the risk of infection in subsequent crops.

In summary, we identified FORL causing FCRR in Hohhot, but there is an urgent need to study FORL in Inner Mongolia for both fresh and processed tomato products. Inner Mongolia is a major tomato production area, having produced 1.1 million tonnes in 2022, accounting for 17.7% of China’s total processed tomato production (6.2 million tonnes, WPTC data). Therefore, it is necessary to provide local growers and workers with more toolboxes for FCRR disease identification and to prevent any potential losses.

## Figures and Tables

**Table 1:** Accession numbers of genes in FORL isolates at NCBI GenBank.

Isolate	ITS	TEF-*1α*	pgx4
T36-2B1	OP600555	OP615672	OP615676
T36-1f	OP600556	OP615673	OP615677
T36-1D	PQ084700	PQ127019	PQ127023
T38-1B	PQ084701	PQ127020	PQ127024
T38-2C	PQ084702	PQ127021	PQ127025
3-15B1	PQ084703	PQ127022	PQ127026

**Table 2:** Pathogenicity of *Fusarium oxysporum* f. sp. *radicis-lycopersici* isolates.

Scientific Classification of Plant Species	Common Name	Leaf Stage	Isolate *	
			T36-1f	T36-2B1
Solanaceae				
*Solanum melongena* L.	Eggplant	4–5	*	*
*Capsicum annuum* L.	Chilli pepper	4–5	+	+
Leguminosae				
*Cicer arietinum* L.	Chickpea	7–8	*	*

* Rating system: * = isolate of FORL recovered from roots; lesions large and roots severely affected; + = isolate of FORL recovered from roots; lesions small, with only a slight discoloration of the roots.

**Table 3:** Sensitivity tests of FORL isolates to seven fungicides.

Isolate	Fungicide *	EC_50_ Values/μg·mL^−1^
T36-1f	97% Hymexazol	139.308
T38-2C		275.158
T36-1f	97% Tetramethylthiuram disulphide	15.142
T38-2C		11.922
T36-1f	98% Pyraclostrobin	1.2982
T38-2C		0.4681
T36-1f	98% Azoxystrobin	1.520
T38-2C		1.0980
T36-1f	97% Tebuconazole	0.038
T38-2C		0.0511
T36-1f	98% Prochloraz	0.005
T38-2C		0.009
T36-1f	98% Thiophanate-methyl	9.875
T38-2C		9.490

* The number indicates purity of fungicides.

**Table 4:** Resistance evaluation of tomato germplasms to *Fusarium oxysporum* f. sp. *radicis*-*lycopersici* T36-1f.

Germplasm	Disease Index (DI)/%	RT ^1^	Germplasm	Disease Index (DI)/%	RT
Y156	62.5 ± 3.5	HS	B63	60.0 ± 7.1	S
Y155	57.5 ± 3.5	S	B64	45.0 ± 0.0	S
Y157	55.0 ± 0.0	S	B51	45.0 ± 7.1	S
Y530	53.1 ± 4.4	S	B77	61.1 ± 5.6	HS
Y61	66.9 ± 2.7	HS	242	72.5 ± 3.5	HS
151-134	27.5 ± 3.5	MR	267	52.5 ± 10.6	S
151-14	30.1 ± 3.3	MS	287	65.0 ± 0.0	HS
151-18	47.5 ± 10.6	S	311	60.0 ± 7.1	S
62	67.5 ± 7.1	HS	309	67.5 ± 3.5	HS
207	72.5 ± 0.0	HS	A3	61.3 ± 1.8	HS
216	55.0 ± 0.0	S	283	57.5 ± 3.5	S
233	55.0 ± 7.1	S	CM 966	37.5 ± 3.5	MS
354	60.0 ± 7.1	S	Caomeifanqie	35.0 ± 7.1	MS
351	52.5 ± 3.5	S	Lüluocheng	45.0 ± 0.0	S
359	61.1 ± 5.6	HS	Hongniuxin	37.5 ± 3.5	MS
Neifan 401	15.0 ± 3.5	R	Shengshijinyu	36.3 ± 1.8	MS
224	47.5 ± 3.5	S	Huangyuanshuai	31.3 ± 2.9	MS
B37	65.0 ± 7.1	HS	Shengfen 88	33.3 ± 5.9	MS
229	42.5 ± 3.5	S	Tianmeiyu	37.5 ± 3.5	MS
236	25.0 ± 0.0	MR	Hezuo918	56.3 ± 2.9	S
B66	78.6 ± 0.0	HS	Shuiguofanqie	34.8 ± 3.8	MS
B53	70.0 ± 0.0	HS	Caomeifanqie-2	40.0 ± 0.0	MS

^1^ RT indicates resistance type, including R = resistant (0.1 ≤ DI ≤ 20.0), MR = moderately resistant (20.1 ≤ DI ≤ 30.0), MS = moderately susceptible (30.1 ≤ DI ≤ 40.0), S = susceptible (40.1 ≤ DI ≤ 60.0), and HS = highly susceptible (DI > 60.1) [16].

## Data Availability

The original contributions presented in the study are included in the article/Supplementary Materials, further inquiries can be directed to the corresponding authors.

## Supplementary Materials

The following supporting information can be downloaded at: https://www.mdpi.com/article/10.3390/jof10090622/s1, Table S1: Concentrations of fungicides in PDA for sensitivity tests; Table S2: The tomato germplasms used in this study.
