# Supplementary material for: Characterisation of Fusarium oxysporum f. sp. radicis-lycopersici in Infected Tomatoes in Inner Mongolia, China
Source: J Fungi (Basel). 2024 Aug 30;10(9):622. doi: 10.3390/jof10090622 (PMC11433067; doi:10.3390/jof10090622)
Supplement: Supplementary file 1 [file jof-10-00622-s001.zip › jof-3100733-supplementary.pdf]

Table S1. Concentrations of fungicides in PDA for sensitivity tests

Fungicide[1]	Final concentration /μg·mL^{-1}
97% Hymexazol	0.0、2.0、4.0、8.0、16.0、32.0、64.0
97% Tetramethylthiuram disulfide	0.0、1.0、2.0、4.0、8.0、16.0、32.0
98% Pyraclostrobin	0.0、0.125、0.25、0.50、1.0、2.0、4.0
98% Azoxystrobin	0.0、0.125、0.25、0.50、1.0、2.0、4.0
97% Tebuconazole	0.0、0.01、0.02、0.04、0.08、0.16、0.32
98% Prochloraz	0.0、0.01、0.02、0.04、0.08、0.16、0.32
98% Thiophanate-methyl	0.0、1.0、2.0、4.0、8.0、16.0、32.0

[1] The number indicates purity of fungicides.

Table S2. The tomato germplasms used in this study.

Germplasm	Type [1]	Generation [2]	Germplasm	Source	Generation
Y156	BM	IL	B63	BM	H
Y155	BM	IL	B64	BM	H
Y157	BM	IL	B51	BM	H
Y530	BM	IL	B77	BM	IL
Y61	BM	IL	242	BM	F1
151-134	BM	IL	267	BM	F1
151-14	BM	IL	287	BM	F1
151-18	BM	IL	311	BM	IL
62	BM	IL	309	BM	IL
207	BM	IL	A3	BM	IL
216	BM	IL	283	BM	IL
233	BM	IL	CM 966	CV	H
354	BM	IL	Caomeifanqie	CV	H
351	BM	IL	Lvluocheng	CV	H
359	BM	IL	Hongniuxin	CV	H
Neifan 401	BM	H	Shengshijinyu	CV	H
224	BM	IL	Huangyuanshuai	CV	H
B37	BM	IL	Shengfen 88	CV	H
229	BM	IL	Tianmeiyu	CV	H
236	BM	IL	Hezuo918	CV	H
B66	BM	H	Shuiguofanqie	CV	H
B53	BM	H	Caomeifanqie -2	CV	H

[1] BM indicates breeding materials provided by Tomato Breeding and Cultivation Research Group of the Inner Mongolia Academy of Agricultural and Animal Husbandry Sciences; CV indicates commercial varieties bought from Inner Mongolia Mengmiao breeding; The number indicates purity of fungicides; [2] IL and H indicate inbred line and hybrid, respectively.
